# COVCOG: Immediate and long-term cognitive improvement after cognitive versus emotion management psychoeducation programs - a randomized trial in covid patients with neuropsychological difficulties

**DOI:** 10.1186/s12883-023-03346-9

**Published:** 2023-08-18

**Authors:** Sylvie Willems, Vincent Didone, Carmen Cabello Fernandez, Gael Delrue, Hichem Slama, Patrick Fery, Julien Goin, Clara Della Libera, COVCOG Group, Michel Moutschen, Anne-Françoise Rousseau, Gilles Dupuis, Maud Billet, Maëlle Charonitis, Valentine Demoulin, Marie Dethier, Camille Guillemin, Fanny Kreusch, Fréderique Leens, Christina Léonard, Alexia Lesoinne, Florence Requier, Mathilde Reyt, Marianne Rotsaert, Fabienne Collette

**Affiliations:** 1 Psychology and Neuroscience of Cognition Unit, Université de Liège, Place Des Orateurs, 1, B33 4000 Liège, Belgium; 2 University Psychology and Speech Therapy Clinic, CPLU, Université de Liège, Liège, Belgium; 3 Clinical Neuropsychological Unit, Liège University Hospital, CHU de Liège, Liège, Belgium; 4 Clinical Neuropsychological Unit, Brussel University Hospital, Erasme, Brussels, Belgium; 5 GIGA-CRC, Université de Liège and Belgian National Fund for Scientific Research, In Vivo Imaging, Liège, Belgium

**Keywords:** Long COVID, Post-acute COVID, Psychoeducation, Cognitive impairment, Cognitive rehabilitation, Counseling, Cognitive behavior counseling

## Abstract

**Background:**

Cognitive difficulties are a frequent complaint in long COVID and persist for more than a year post- infection. There is a lack of evidence-based data on effective intervention strategies. Non-pharmacological intervention programs that are used with other neurological populations have not yet been the subject of controlled trials. COVCOG is a multicentric, randomized trial comparing cognitive intervention and a cognitive-behavioural counselling.

**Methods/design:**

Patients with long covid are selected and recruited at least three months post-infection. Patients are randomised in a 1:1 ratio into the cognitive (neuropsychological psychoeducation) and affective (emotion management with cognitive-behavioural counselling) intervention arms. The inclusion of 130 patients is planned. The cognitive intervention includes psycho-educational modules on fatigue and sleep, attention and working memory, executive functions and long-term memory. The affective intervention includes modules on emotion recognition and communication, uncertainty management and behavioral activation. The main objective is to reduce cognitive complaints 2 months after the intervention. A Follow-up is also planned at 8 months.

**Discussion:**

Given the long-term effects of Covid on cognition and the negative effects of cognitive impairment on quality of life and social participation, it is important to determine whether low-dose, non-pharmacological interventions can be effective. The trial will determine which of the usual types of intervention is the most effective.

**Trial registration:**

Clinicaltrials.gov Number: NCT05167266 (21/12/ 2021).

## Introduction

### Background and rationale

It is now acknowledged that the disease caused by SARS-CoV-2 infection, COVID-19, is a multisystemic syndrome [1] affecting several organs beyond the respiratory system, including the brain [2]. As a result, cognitive difficulties are regularly observed, sometimes with a predominance of deficits in executive functions, attention and processing speed, as well as memory problems [3, 4] sometimes more pronounced in verbal modality, and sometimes even language disorders [5]. Many studies also showed that neurological symptoms [6], including these cognitive disorders and mental fatigue, could persist for several months after the acute infection phase. This persistent syndrome called "Long COVID" has been observed after long delays (e.g., after 1 year) [7] with important consequences for quality of life [8].

These difficulties appear to have a multifactorial origin. An association is observed between these cognitive deficits and alterations in olfaction and taste [7], which may support the implication of central nervous system involvement. Neurobiological pathways by which SARS-CoV-2 infection may generate or exacerbate these disorders include direct viral encephalitis, cytokine-related neuroinflammation, coagulopathy, vascular endothelial dysfunction and antineuronal antibody production, or cerebral microvascular injury, with a possible co-occurrence [9–11]. The hypothesis of the effect of hypoxia on the hippocampus has also been discussed. This hypothesis is supported by, among others, the observed association between memory deficits and respiratory distress during the acute phase [7]. In parallel, brain imaging studies have demonstrated hypometabolism in the fronto-parietal [12], dorsolateral prefrontal [13], superior temporal, precentral and lateral occipital regions [14]. Based on a review of existing data, Toniolo et al. [15] proposed that SARS-CoV-2 could preferentially and directly affect the frontal lobes and/or frontal networks. A deficit in glutamatergic neurotransmission was also observed with spectroscopy [16].

Regarding the prevalence of persistent cognitive difficulties, a meta-analysis by Ceban et al. [17] of 74 meta-analyses reported a proportion of 22% 3 months or more after confirmation of infection (95% confidence interval 17%-28%). The prevalence increased to more than one-third in studies that objectively assessed the disorder using neuropsychological assessment. Similar incidences of fatigue and cognitive impairment were observed among hospitalized and non-hospitalized populations, regardless of the time after the acute phase (< 6 versus ≥ 6 months). This latter observation contrasts with other neurological disorders (such as anosmia) that tend to decrease over time. Finally, several studies have explored the prevalence of deficits on neuropsychological tasks in patients with complaints still present 4 months after infection. They showed a prevalence of deficits higher than 50% [3, 7], reaching up to 75% [8]. These long-term difficulties still mainly concern processing speed and attentional functions, followed by memory in verbal and visual-spatial modalities [3, 5, 7]. A progressive positive but very gradual evolution is however noted after one year [7] and even later than 16 months after the acute phase [18], mainly for verbal memory but also for attentional and executive tests. It is not yet known whether spontaneous recovery will be complete. In this context, it is important to consider possible interventions.

Cognitive remediation therapy has been shown to be effective in reducing long-term cognitive deficits or their impact on daily life in several other neurological conditions [19–22]. To our knowledge, existing data in the long covid are still poor. However, a case–control study [23] compared a small group of patients who received a 6-session intervention consisting of cognitive exercises and counseling (*n* = 15) to a group of control patients without treatment. The intervention improved performance on cognitive tasks, with a correlation to measures of quality of life. Given the lack of validated interventions to reduce post-COVID cognitive symptoms, our goal is to also evaluate the effectiveness of a brief, multidimensional cognitive psychoeducation intervention targeting 4 symptoms frequently reported in patients with COVID: fatigue, attention, memory, and executive functions. This intervention will be compared to cognitive-behavioural counselling (CBC) and psychoeducation, which is another widely used approach [24] that can have positive effects for patients with cognitive difficulties [25, 26]. This type of intervention could therefore be another avenue for long covid. Furthermore, in the longitudinal study by Diana et al. [18], more than 30% of patients were found to have some degree of depressive and PTSD-related symptoms more than one year after acute recovery. Correlations between psycho-affective and cognitive measures were observed indicating the impact of affective state on cognitive functioning. Taken together, these results suggest that an intervention targeting affective difficulties (anxiety management, etc.) could also have a positive effect on patients' well-being.

### Objectives

In this context, our clinical trial aims to explore the potential effectiveness on cognitive complaints of two common low-dose interventions, one targeting cognitive difficulties (cognitive intervention) and the other targeting affective difficulties that may enhance cognitive difficulties, with a CBC approach (affective intervention). This trial will provide information on these interventions and their feasibility. It will allow us to explore the expected superiority of the cognitive intervention over the affective one.

### Trial design

COVCOG is a multicenter, randomized controlled proof-of-concept trial conducted with two parallel groups (ratio 1:1) comparing psychoeducation interventions that focus on cognitive (*n* = 65) versus affective (*n* = 65) difficulties. Randomization with minimization will be used.

## Methods

Reporting and methodology for the proposed study follow the Standard Protocol Items Recommendations for Interventional Trials (SPIRIT). The protocol has been registered on Clinicaltrials.gov: NCT05167266 (21/12/ 2021).

### Study setting

The trial is conducted by the University of Liege in Belgium. The data are collected in Belgium at four centers: the university of Liège (the Psychological Clinic of University of Liege, CPLU and the university hospital, CHU-Liège), the university hospital of the University of Brussel (ERASME hospital), the regional hospital of Liege (CHR-Liège), and the catholic hospital of Liege (CHC-Liège).

### Eligibility criteria

All volunteers will be screened by a psychologist. To be included in the study, participants must meet the following criteria: be able to understand the information and consent forms; aged 18 to 70 years; medically stable and at least 3 months after a positive COVID 19 infection confirmed either by PCR, self-test or medical advice; report sufficiently good physical condition to attend the appointments; report no major hearing or vision disorders; report cognitive complaints (that place the person in the top 20% of dissatisfied functioning on the BRIEF or MMQ questionnaires); poor but not necessarily deficient objective performance (supported by a score below the 20th percentile on one task of the cognitive battery). Participants will be excluded if they have any chronic or remote neurological disorder (i.e. stroke, head trauma, epilepsy, tumor); preexisting cognitive impairment (associated with another minor or major neurocognitive disorder; intellectual disability); acute brain injury or acute encephalopathy from another etiology than covid (e.g., sepsis, liver or renal failure, alcohol or drug withdrawal, drug toxicity); documented preexisting history of psychiatric illness (including substance abuse); open-heart cardiac surgery or cardiac arrest during the last 6 months; current hospitalization; current revalidation care with cognitive treatment. Participants with preexisting neurological, cognitive, or psychiatric disorders (i.e., preceding COVID) are also excluded. Individuals with disorders co-occurring with COVID are not excluded.

### Informed consent

Researchers who take consent from participants are psychologists who are certified in good clinical practice and trained by the principal investigator. The research project is conducted with approval by the relevant Ethic Committees, with the Hospital-Faculty Ethics Committee of CHU Liège serving as the Central Ethic Committee (number: 707).

### Interventions

#### Explanation for the choice of comparator

Given that it would have been unethical not to propose an intervention, a pragmatic clinical trial logic was adopted by comparing the effect of cognitive psychoeducation program with another psychoeducation program targeting affective difficulties, two common approaches in current practice. We based our reasoning for choosing the second intervention on two elements. Psychological difficulties are also observed in Long-COVID patients [27]. An intervention targeting affective difficulties (anxiety management, etc.) could therefore have at least an effect on patients' well-being. Second, several studies indicate that a treatment targeting these affective dimensions even in a brief psycho-educational format could be effective for neurological populations with different symptomatology, including also mental fatigue and cognitive difficulties [25, 26].

#### Interventions description

The cognitive and affective interventions are a 4-session, psycho-educative interventions designed to prevent post-acute cognitive symptoms. Each individual session will last 90 min. One month after the last session, a reactivation session of 30 min is organized (remotely or in person) to reactivate or help the patient to apply one or the other strategy in his daily life.

##### Cognitive intervention

The structure of the four sessions is similar: 1) explanation of cognitive (dys)functioning; 2) identification of significant problems in the daily life of each participant; 3) explanation and application of (meta)cognitive strategies. Throughout the presentation, the risks of causal over-attribution (i.e., misattributing common difficulties in daily life to COVID) and the anticipation of long-term negative outcomes will be addressed. The patient will also be made aware of the coping style that can aggravate difficulties (passivity, activity avoidance, focus on difficulties).

Each of the four modules concerns a specific cognitive domain. A module can be delivered in one session but may take less or more than one session depending on the patient's difficulties. The content of the modules is also accessible outside the sessions via a video support. The patient is invited to explore these contents between sessions.

#### Module 1—Cognition in COVID; sleep and fatigue

The first step is to provide feedback on the results of cognitive assessment from the baseline visit and to relate it to the cognitive deficits observed in the long covid. Validating and normalizing symptomatology while simultaneously highlighting appropriate strategies to alleviate problems is an important step in psychoeducational programs [28].

Regarding sleep and fatigue, the mechanisms underlying sleep–wake regulation are explained to the patient. For fatigue management, recommendations include the use of a fatigue diary to identify triggers and patterns of fatigue, reorganization of patient’s schedule of the week to avoid exhaustion, practice of physical activity as well as relaxation techniques.

#### Module 2—Working memory and attentional functions

Psychoeducation focuses on 1) how to optimize the environment to minimize difficulties (how to reduce interference and dual-task situations, etc.); 2) how to optimize task design to minimize difficulties. If necessary, Time Pressure Management (TPM) is also used to improve adaptation to slowed information processing [29, 30].

#### Module 3 – Executive control

Regarding executive functions, the literature on mild cognitive impairment supports the use of instructional procedures for training patients to regulate their behavior and thinking (see DCoE and DVBIC consensus conference) [31]. Thus, the procedures for teaching metacognitive strategies include self-instructional learning aimed at improving 1) the formation of goals relevant to daily needs, 2) the planning of strategies to achieve these goals, 3) the monitoring of performance and the adaptation of the strategy, if necessary, 4) the evaluation of the achievement of the set goals. Concretely, Levine et al.’s *goal management training self-instruction for patients* are used with the classical five-step training [32, 33]. Different advices will again be given, such as the need to automate procedures, to do only one thing at a time, to decrease the sources of distractions, to know one's time and the amount of effort.

#### Module 4 – Long-term memory

Practice-standards for mild memory impairments suggest the use of internalized strategies (e.g., mental imagery) and external memory compensations (e.g., notebook, diary) to enhance retrieval of information. Training the use of these types of memory compensations and aids within activities of daily living continues to be efficacious and consistently supported by empirical evidence [34]. In this context, psychoeducation will then focus on two areas: 1) External aids; 2) Internal strategies. Regarding external aids, advices will be offered on how to relieve memory and especially prospective memory with the use of mobile phone and diary [33, 35]. Regarding internal strategies, the clinician will teach participants to use self-initiated strategies in situations requiring episodic memory. Other global techniques will be explained to help memorization [21].

##### Affective intervention

The structure of the four sessions will be similar: 1) analysis of a fictitious vignette illustrating the thoughts and affect that can be provoked by the long COVID; 2) in connection with the explanations, identification of significant problems in the daily life of each participant; 3) explanation of psychological functioning; 4) discovery and application of strategies. Each time, the strategies will have to be practiced at home and will be discussed again at the next session.

#### Module 1—Recognizing emotions and affective states

Psychoeducation on emotions will be proposed (e.g., triggers of emotions, category of primary and secondary emotions, meaning of emotions). The patient will be asked to reflect on his or her emotions associated with long-covid and their manifestations (internal and external physical and physiological manifestations; cognitive effects of emotions and thought patterns; subjective feeling; action tendencies). The effects of negative emotions will also be discussed. The Stimulus-Organismic-Response-Consequence model (SORC) [36] will be taught and used as a basis for home observation with diary. Finally, body awareness/relaxation exercises will be proposed. The respiration method will be taught.

#### Module 2—Accepting and communicating about difficulties

After a review of the patient's diary, the patient will be asked to share his or her observations on the emotion self-observation. Afterwards, the interest of being able to communicate calmly about one's cognitive difficulties and one's feelings in relation to the COVID will be discussed with the patient. The principles of assertive communication will be addressed by following Fanget and Rouchouse's principles [37] and will be applied to different situations. Finally, body scan exercises will be proposed.

#### Module 3—Accepting the uncertainty associated with difficulties

Following Ladouceur's model of uncertainty management [38], the patient will be guided to recognize the elements of uncertainty intolerance (identification of worries; inhibition related to anxiety and/or problem-solving difficulty; avoidance/neutralization; intolerance of uncertainty; exhaustion). Overestimation of the usefulness of worries will be addressed. The patient and clinician will then discuss an uncertainty exposure exercise related to cognitive difficulties.

#### Module 4—Behavioral activation

Patients will be invited to identify their values to better calibrate the choice of activities according to their fatigue but also according to what is important to them. If the patient has difficulties with ruminations, the impact of these on activation will be discussed. A behavioral activation plan will also be constructed with the patient. Different techniques will be taught (e.g., increasing rewarding activities, recalling positive moments of the day, mental imagery of positive events).

### Criteria for discontinuing or modifying allocated interventions

Reasons for discontinuing protocol treatment may include, but are not limited to, the patient's desire not to continue or a change in health status requiring alternative treatment. There are no reasons envisaged for a change of allocation.

### Strategies to improve adherence to interventions

Clinicians are trained in psychoeducation interventions; manuals list each module ingredient in detail (with examples of instructions and exercises to be provided to patients). A roadmap summarizes the sequence of steps in each module. An adherence checklist with the essential topics to be covered is to be completed by the clinician. Each deviation will be noted in the eCRF.

### Relevant concomitant care permitted or prohibited during the trial

Treatment similar to that of one of the two arms is not accepted.

### Provisions for post-trial care

Not applicable.

### Baseline and endline assessment

A psychological evaluation is administered before and 2 months and 8 months after treatment. This evaluation involves 2 sessions of 90 min.

#### Primary outcome

The primary outcome is a subjective report of difficulties experienced by patients in daily life at two months post-intervention measured via two questionnaires.

The Behavioral Rating Inventory of Executive Function (BRIEF) is a validated questionnaire assessing the main cognitive complaints of the patients (e.g., attention, concentration, disorganization,). It measures the impact of difficulties in daily life and has two indexes (Behavioral Regulation, BRI; Metacognition, MI) and an overall score (Global Executive Composite, GEC). The BRIEF’s reliability is high; Cronbach’s alphas for the BRI and MI have been found to be 0.94 and 0.96, respectively [39].

The Multifactorial Memory Questionnaire (MMQ) is a validated questionnaire measuring affects related to memory abilities, frequency of forgetfulness in different situations, and strategies used in everyday life to cope with memory difficulties. The scores proved to be reliable (Cronbach's α for the subscales ranged from 0.79 to 0.88) [40].

#### Secondary outcome 

BRIEF and MMQ will also be administered 8 months after intervention. Other secondary outcomes at 2- and 8-months post-intervention are [1] quality of life; [2] the presence of objective cognitive difficulties, assessed by neuropsychological tasks; [3] fatigue level and sleep quality; [4] psychological distress; [5] work productivity and activity impairment.

##### Quality of life (QOL)

The status of Quality of life is self-assessed through the Quality of Life Systemic Inventory [41] which measures QOL through three distinct scores: the gap score (the difference between ‘desired situation’ and ‘current situation’), the goal score (the difference between the desired situation and the ideal situation) and the rank score (the importance given to the item or situation). This tool has a good test-rest reliability (0.86).

Participants also complete the EQ-5D [42], a standardized measure of health-related quality of life developed by the EuroQol group to provide a simple, generic questionnaire for use in clinical and economic assessments and population health surveys [www.euroqol.org].

##### Neuropsychological tasks

A cognitive assessment is carried out by a neuropsychologist before the intervention as well as 2 months and 8 months after the intervention. The battery takes about 150 min to complete and is administered in two sessions. It includes several psychometric tests with normative data for french-speaking population.

The tasks chosen to detect the presence of objective cognitive difficulties in the attentional and executive domains are the following: selective auditory and visual attention, divided attention, updating and flexibility tasks from the Attentional Performance Battery (TAP, v2.3.1) [43], the D2-R task assessing concentration skills [44]; the Stroop test assessing inhibition [45]; phonemic and semantic fluency [45] and the Brown-Peterson task, also assessing divided attention [46]. Long-term memory tasks are the Word list subtask from the RBANS [47] and the Brief Visuospatial Memory Test-Revised [48].

Global cognitive performance is assessed with the screening tool Montréal Cognitive Assessment (MOCA) which is widely used in clinical settings and research [49].

##### Fatigue and sleep

A 19-item self-report inventory (Pittsburgh Sleep Quality Inventory, PSQI [50] is used to assess sleep quality and disturbances over a 1-month interval. The psychometric and clinical properties of the PSQI have been proven in the past and confirm its utility both in clinical practice and research activities. PSQI has a good internal consistency (Cronbach's α = 0.83).

Fatigue level was also examined through the Modified Fatigue Impact Scale (M-FIS). The M-FIS is a 21-item self-report questionnaire developed by the US NMSS (National Multiple Sclerosis Society) and derived from the original 40-item Fatigue Scale [51]. This questionnaire is used to assess the impact of fatigue experienced in daily living during the past 4 weeks from which cognitive (cMFIS), physical (pMFIS) and psychosocial fatigue scores (psMFIS) can be derived. The M-FIS has a correct Cronbach’s α of 0.80.

##### Psychological distress

The Outcome Questionnaire 45 (OQ-45) [52] is a 45-item self-report inventory used to measure psychological change from which Symptom Distress, Interpersonal Relations, Social Role scores can be derived. The OQ-45 has high reliability and internal consistency (Cronbach's α = 0.93) [52].

##### Work and activity

The Work Productivity and Activity Impairment (WPAI) [53], 6-item questionnaire is a well validated instrument to measure impairments due to overall health and symptoms in work and activities.

### Participant timeline

**Fig. 1:**
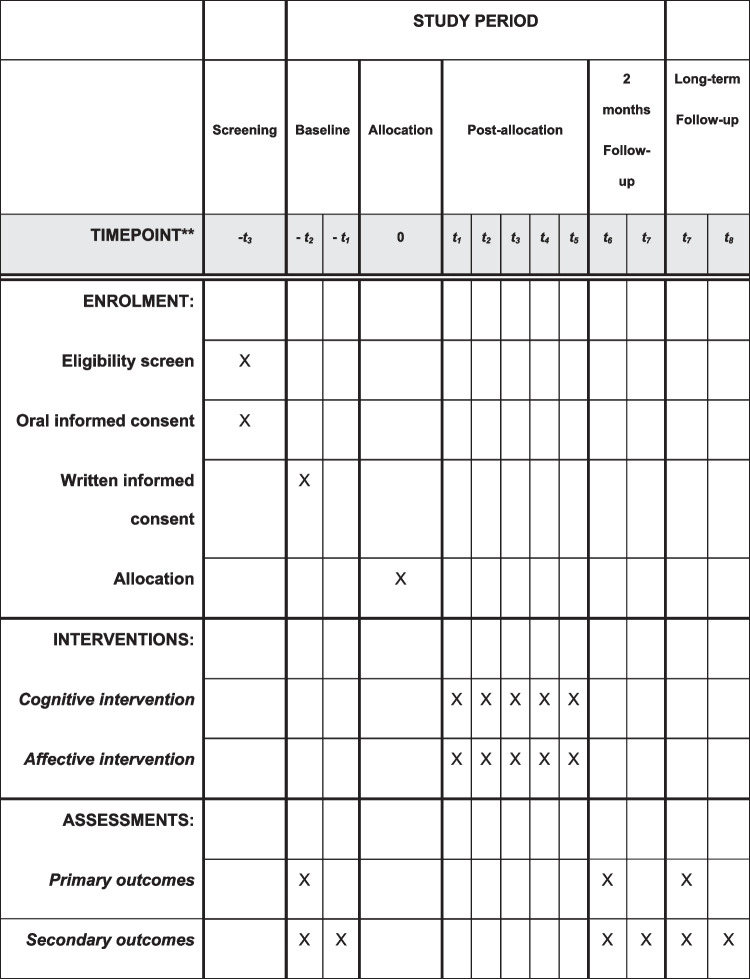
Participant timeline

After a telephone screening, the patients corresponding to the eligibility criteria are invited to participate in the study with a first appointment for the baseline. The full assessment protocol is then administered. Individuals completing the inclusion criteria are randomized within one week of baseline and intervention can start the following week. The intervention sessions are proposed with a week interval followed by a reactivation session 1 month after the 4th session. Immediate and long-term follow-up will be administered 2 and 8 months after the end of intervention. This timeline is summarized in Fig. 1.

### Sample size

The uniqueness of our research does not make it possible to determine the exact expected effect size. Thus, we decided to follow a proof-of-concept study logic with a pragmatic approach to setting our sample size. A feasibility analysis led us to the conclusion that we could reach a sample of 130 participants in 10 months. We next measured the sensitivity of our experimental design on this expected recruitment rate with Gpower [54] based on the following values: alpha corrected for 2 primary outcomes equal to 0.025, power equal to 0.80, experimental design corresponding to a mixed analysis of variance with repeated measures and an intergroup variable, with an intra-subject correlation of 0.5. Results from the sensitivity analysis show that our experimental design will be able to detect small effect sizes (interaction effect in a mixed anova) of f = 0.137.

### Recruitment

An initial set of patients will be identified by the usual physicians/clinical teams of the centers participating in the study. Patients will be informed and will contact the study team. Participants will also be recruited by word of mouth ("flyer", Covid long facebook group, primary care physicians). Finally, if needed, patients who had a post-Covid follow-up consultation in one of the centers will be contacted again.

### Assignment of interventions: allocation

#### Sequence generation

Given our small sample size, and because individuals with more severe cognitive impairments might respond differently to the intervention than those with milder difficulties, we will use randomization by minimization. Although severity and interval since COVID episode are probably important prognostic factors in outcomes, we decided to achieve trial arm balance only on age, education level, gender, Centre, and severity of cognitive deficits and complaints. For cognitive deficits, scores will first be Z-scored based on normative data, and next a global Z-score calculated on all variables will be considered (sum of Z-scores divided by number of measures). A Z-score will also be calculated for both tools assessing cognitive complaints. For both these objective and subjective indices, balance will be done by Z-score range of 0.5 (e.g., Z-score from 0 to -0.50; -0.51 to -1, etc.). For age we will use 18–29; 30–39; 40–49; 50–59; 60–70. For education, we will use 4 levels: 1. primary or professional education; 2. secondary education; 3. bachelor; 4. master. In the calculation of the imbalance each arm and each prognostic factor will have the same weight. The imbalance score is calculated based on all previous allocations as well as the current participant's hypothetical allocation to each treatment. The distance measure used to calculate the imbalance score is the marginal balance. If there is no imbalance, the allocation will be completely random. If there is an imbalance, the treatment with the lowest imbalance score is chosen with a 75% probability.

#### Concealment mechanism and Implementation

After the baseline session, an attribution request is then emailed, including the ID, to a researcher responsible for the attribution process with the QMinim software. This researcher does not participate in the follow-up assessment. This researcher then sets up the appointment with the clinician of the assigned arm.

### Assignment of interventions: Blinding

#### Who will be blinded

The statistician will be blind to the content of the intervention arm that will be called “A” and “B”. The neuropsychologists who perform the baseline assessment do not know about the randomisation (which is only carried out after the visits). The neuropsychologists that will be in charge of follow-up will also be blinded to the arm of the intervention. Patients will be warned to not mention their intervention arm or provide information that could cue the clinician (this will be reminded before each assessment session). If the clinician becomes unblinded during the testing session, this will be reported in the eCRF.

The person administering the intervention cannot be blinded, as the content is different. The participants cannot be blinded. However, the trial will be presented as “assessing the positive effect of two interventions on post-covid cognitive difficulties” Our prediction (superiority of the cognitive intervention) will not be communicated to the participants until the end of the trial (last follow-up for the last participant included).

#### Procedure for unblinding if needed

Not applicable: the patients and the clinicians know the intervention as the content of the sessions is different in the two arms.

### Data collection and management

#### Plans for assessment and collection of outcomes

All tests and questionnaires are administered by a trained psychologist specialized in neuropsychology. The primary outcome is administered via a computerized questionnaire system in the eCRF (Castor). MMQ and BRIEF (primary outcome) will be remotely administered again after a 2-week interval to take account of fluctuating difficulties.

#### Plans to promote participant retention and complete follow-up

Patients will be reminded by the researcher to log in and complete assessments.

#### Data management

The data are entered into the eCRF by the clinician researchers in charge of the evaluation. A manual was written to guide the encoding. Double entries were made on the first patients. 100% of the primary and 10% of the secondary outcomes are verified by independent monitor of Antwerpen University Hospital Clinical Trial Centre (UZA CTC). Last checks for data values will be done by the statistician and the data manager of UZA CTC.

#### Confidentiality

Confidentiality and data protection will comply with European and Belgian directives on the protection of individuals with regard to the processing of personal data and on the free movement of such data (GDPR). Standard operating procedure (SOP) for data protection are detailed on the website of the Faculty of Psychology EC (https://www.fplse.uliege.be/cms/c_4511361/fr/psy-comite-d-ethique). We will benefit from the procedures developed by the Data Protection Officers (DPO) at Liège university and CHU.

Personal information and linking code will be saved in a location separate from the one where data were collected, in encrypted, password-protected folders. Access to these data will be limited to only those who need it for the purposes of trial management, quality control, audit, and analysis. These data will be destroyed 5 years after the end of the trial.

Data entered into the eCRF will be coded and pseudo-anonymized. Computers used for storage of personal and research data are professional (not personal) laptops, protected by password. The folders where information will be stored will be also protected by password, and temporarily backuped on an encrypted external device stored in the researcher office.

#### Plans for collection, laboratory evaluation and storage of biological specimens for genetic or molecular analysis in this trial/future use

Not applicable for this study.

## Statistical methods

### Statistical methods for primary and secondary outcomes

#### Summary of baseline data and flow of patients

Preliminary analyses will be conducted to ensure comparability of both groups on demographic (sex, age, education years) and clinical characteristics at baseline. Two-sided independent sample t tests will be conducted for continuous variables and categorical variables will be analyzed with Fisher exact tests. A consort flow diagram will be produced to get an overview of the number of patients available at each stage: eligibility, allocation, discontinuation, short and long-term follow-up.

#### Primary outcome analysis

Primary outcome will be first analyzed on an intention-to-treat (ITT) basis, including all patients as originally allocated after randomisation. For missing information due to patient drop-out, we will rely on the linear mixed model and consider the baseline data as part of the outcome matrix. We will also perform a per-protocol (PP) sensitivity analysis including only those patients who completed the treatment originally allocated.

Analyses should be considered as hypotheses generating. To explore the effects of the intervention type on the two primary outcome measures, the results will be compared using linear mixed models. More precisely, the moment of the measure (baseline and immediate follow-up), the intervention (nested within the centers), the centers and the interaction of these factors will be considered as fixed factors. A nested random intercepts and slopes for each participant within the centers will be implemented. Age groups, educational level, objective Z-score (Z-score from 0 to -0.50; -0.51 to -1, etc.) and gender will also be included in the model as stratification factors. Following the linear mixed model, linear contrast will be performed between the two levels of the intervention variable on the first follow-up measure (T1) to explicitly compare the potential benefit of the intervention. Bonferroni adjustment will be performed to correct for multiple testing, more precisely 2 tests (the number of primary measures), and p-value < 0.025 will be considered as statistically significant. 95% CI and standardized effect sizes (SES) from the null model will be calculated. The following convention will be used to interpret effect size: small 0.2–0.49, moderate 0.5–0.79, large ≥ 0.8. As a Proof-of-Concept (POC) study, estimation will be focused on ES but will include p-values as help in interpreting the data.

#### Secondary outcome analysis

Mixed linear models will also be used to assess the maintenance of effect across time (baseline, immediate and long-term follow-up). We will re-run the same analysis as for primary outcome, this time by including scores at long-term follow-up. Intervention effect size will be estimated using the same mixed models for each secondary and exploratory outcome measurement (Scores of ISQV and EQ-5D, the 5 overall Z-score per cognitive domain, Outcome-Questionnaire 45, PSQI, M-FIS) at both short-term follow-up (T1) and long-term follow-up (T2). As a rule, stratification factors will be included in the analyses.

### Interim analyses

The objective of interim analyses will be to determine if there exist profiles of patients with specific characteristics, to assess if the interventions act differently according to subgroup. We hypothesize a subdivision of patients according to the presence of a more cognitive or psychological profile. For this Latent Profile Analysis (LPA), the z-standardized mean scale scores of the following variables will be entered in the model: composite scores for memory, attention and executive performance, global score at OCQ and M-FIS. All this continuous variables will be used If the presence of these two sub-groups is confirmed, we will next assess if they differ on primary outcomes (complaints at BRIEF and MMQ scales), quality of life (ISQV), medical variables (length of potential hospitalization; presence and duration of ventilation; presence of neurological symptoms at acute phase time since infection), presence/absence of antecedent psychological difficulties, age and sex.

### Methods for additional analyses (e.g. subgroup analyses)

Analyses will be conducted to identify trajectories of participants according to their baseline characteristics (i.e., subgroup identified with the cluster analysis) and the allocated intervention. To explore the effects of the intervention on the two primary outcome measures according to the baseline participant characteristics (profile corresponding to cognitive or psychological difficulties), the same analysis will be performed as for primary outcomes (linear mixed models, see (Statistical methods for primary and secondary outcomes), with inclusion of the subgroup as a supplementary fixed variable.

Analyses will be conducted to identify individual trajectories, in the different cognitive domains and for primary outcome, to identify which variable predict long-term cognitive sequelae. Our analyses will include latent growth curve modelling techniques that use repeated measures to estimate trajectories.

### Methods in analysis to handle protocol non-adherence and any statistical methods to handle missing data

In the ITT analysis, we will measure our primary and key secondary outcomes by patient report in the last assessment. For the PP analysis, the proposed linear mixed model allows missing values at certain time points. The missing value assumption of the model is Missing At Random which means that missing values can only be dependent on the observed responses which seems a reasonable assumption in this case. Missing data should be kept at minimum by applying the following rules: We will check completion of questionnaires at the end of the testing session and ask the participant to try to complete missing information.

### Plans to give access to the full protocol, participant level-data and statistical code

The complete protocol is available on the KCE website. (https://kce.fgov.be/fr/kce-trials/essais-cliniques-finances). Data may be available upon reasonable request to the Principal Investigator.

## Oversight and monitoring

### Composition of the coordinating centre and trial steering committee

In summary, ULiège is the study Sponsor. Willems S. and Collette F. are the Chief Investigator (CI) and responsible for clinical elements of the trial. ULiège and UZA CTC are both responsible for project management. ULiège and UZA CTC will hold joint data controller responsibilities.

### Trial Steering Committee (TSC)

A TSC meets three times in the first year and twice in the second. The committee will provide overall supervision of the trial and ensure that the study is conducted according to the protocol and within the overarching ethical framework. It is composed of chief investigators, trial project manager, statistician, clinical experts (Chief of Internal Medicine and Chief of post intensive care Unit) of the coordinating centre; principal investigators of each participating centre, independent clinical expert (psychologist from University of Montreal); representative of UZA CTC; representative of the funder (KCE); two representatives of patients.

### Trial Management Group (TMG)

The TMG is the executive decision-making body and is responsible for the day-to-day running and management of the trial. It is led by the CIs and consists of representative of UZA CTC, representative of the funder, project manager and principal investigator from coordinating centre. The team meets each trimester (or more frequently if needed) via a teleconference to discuss clinical challenges and risks.

### Composition of the data monitoring committee, its role and reporting structure

Charter can be found, if not in the protocol. Alternatively, an explanation of why a DMC is not needed.

Data monitoring is under the responsibility of CTC UZA, that is independent from the sponsor and funder. A Clinical Monitoring Plan (CMP) was conjointly written by the CTC UZA, in collaboration with the chief investigators. The CMP establishes the guidelines for conducting monitoring visits and related tasks for monitoring protocol Long Covid. The CTC UZA will perform monitoring tasks in accordance with the protocol specific requirements. This plan aims to support the UZA monitor(s) to ensure that the investigational sites follow the International Conference on Harmonisation (ICH) Good Clinical Practice Guidelines (GCP), the local regulatory requirements and he study protocol.

### Adverse event reporting and harms

The project does involve interventions, but they are considered ‘low risk’ with no anticipated serious adverse events. Here, we should observe only mild adverse events related to the interventions, such as increased anxio-depressive affects in some patients when faced with cognitive difficulties. Such adverse events will be discussed with clinician and a trial safety group (composed of one principal investigator of the center, chief investigator, Trial Project Manager, one representative of patients) to determine the most appropriate response (e.g. mail to the general practitioner, psychological counselling outside of the intervention protocol). The date, description of the related adverse event, solution proposed will be recorded in the eCRF and in the source documentation (patient medical record). This information will be also transmitted to TSC.

### Frequency and plans for auditing trial conduct

Trial conduct will be closely monitored by independent subgroup (as previously described). An independent audit will be ordered by the funder.

### Plans for communicating important protocol amendments to relevant parties (e.g. trial participants, ethical committees)

Any proposed amendments to the protocol will first be discussed within the Trial Management Group and the Trial Steering Committee. Approval will be required from all parties before implementation (Funder and Ethics Committee). The chief investigators will take responsibility for communicating protocol amendments to all trial members and participating centers.

### Dissemination plans

Scientific results of the project will be disseminated via publications in peer-reviewed journals and in abstracts of national and international conferences. Open access will be guaranteed by filling all publications related to the project in the ULiège institutional repository—ORBI. Decisions about the scientific content and the publisher will be decided conjointly by researchers involved in the publication.

We will rely on patient representatives to ensure that information emanating from the project can be utilized to support information towards that population.

Neurologists, physical therapists, intensive care physician, neuropsychologists, and psychologists, all involved with the medical and social care of patients with cognitive difficulties are targets for dissemination of information about the objectives and results of COV-COG.

## Discussion

This randomized controlled trial with minimization aims to test the effectiveness of two approaches commonly proposed to people with cognitive complaints on patients with long covid. Because of the impact of cognitive disorders on functional disability and long-term quality of life, such clinical trials seem essential to target the type of intervention that can be proposed to reduce post-COVID cognitive disorders and their impact on daily life (subjective perception of difficulties and quality of life). This is especially true given the unknown outcome of long-term cognitive deficits in that population.

The cognitive approach aims at a better understanding of cognitive impairments and the adoption of adequate strategies to manage them. The approach targets difficulties frequently reported in patients with COVID (sleep and fatigue, attention and concentration, memory, and organization) with several modules whose dosage is adapted to the person's difficulties. The intervention is based on a biopsychosocial model (i.e., an integrative approach taking into account cognitive, contextual and psychological factors) and therefore also aims to reassure, reduce some misconceptions and negative interpretation bias of difficulties. This psychoeducational approach is different from restorative cognitive intervention which aims to improve cognitive performance itself. This type of high-dose intervention requires several dozen sessions with specific exercises to train deficient cognitive process [27]. Psychoeducation is a first-line intervention that is less costly in terms of time and money than traditional cognitive interventions. Indeed, cognitive psychoeducation is short (usually less than 10h) and deals on the management of cognitive difficulties in daily life and not on cognitive functioning as such in objective performance tasks. This approach allows patients to regain control of their symptoms by focusing on the controllable parameters related to their complaints. This type of intervention has positive effects in a wide variety of disorders (e.g., mild traumatic brain injury, stroke, etc.) [55].

In the context of mild traumatic brain injury (mTBI), persistent cognitive difficulties can be addressed through this type of cognitive psychoeducation. However, some psychoeducational programs focus less on cognitive difficulties per se and more on managing the negative emotions associated with cognitive difficulties and other post-traumatic symptoms [56]. This approach is easily justified by some data in the literature. In the case of mTBI, the results of numerous meta-analyses suggest that long-term symptoms are increasingly unrelated to the brain injury itself [19]. Over time, there is dissociation between objective measures of cognition and self-reported cognitive complaints. It is now known that the difficulties experienced by the patient, their functional impact and the reduction in quality of life are going to be influenced by a large number of variables, such as sleep and fatigue, but also the patient's perception of its functioning, affective, physical and social factors, etc. [57]. Therefore, while performance on objective tests tends to improve over time in most patients, some individuals experience an increase in distress with a chronicization of difficulties and their functional impact [33]. In long covid patients, Delgado-Alonso et al. [30] observed that the subjective cognitive complaints significantly but moderately correlate with objective cognitive performance (the time since the infection was 9.12 ± 3.46 months). This correlation was no longer found in the Gouraud et al.’s study [58] testing patients one year post-infection. None of the objective neuropsychological test scores was significantly associated with persistent cognitive complaints. This lack of correlation echoes the results observed in the mTBI.

In this context of a potential dissociation between the evolution of complaints and objective difficulties, a CBT type approach also seems appropriate. Therefore, the cognitive intervention is here compared to another potentially effective intervention focused on the management of emotions. It will aim at helping patient to better recognize their emotions, to determine how to react to them, by giving them the tools to better manage their post-COVID anxiety and stress. Patients will also be brought to become aware of their values and to develop his/her motivation to change. Finally, patients will also be made aware of interpretation biases and negative adaptive behavior (e.g., avoidance of problem situations).

It is reasonable to think that the effect on cognitive functioning of an intervention targeting affective dimensions (anxiety, depression, motivation, etc.) will be lower than an intervention more specifically targeting cognitive dimensions. However, this prediction has to be taken with caution. No study at this time assessed which of the two interventions has the larger effect in long covid patients or in other neurological population. Furthermore, studies testing the effectiveness of a CBT approach used very general scales measuring the set of persistent post-concussion symptoms after mTBI. They do not specifically include measures of cognitive complaints. There is evidence in studies that this type of intervention results in moderate changes in quality of life, with small effects on functioning in daily life and neurological complaints [59]. On this basis, it is difficult to conclude whether these interventions have a direct effect on cognitive complaints specifically.

To the best of our knowledge, there is no clinical trial dealing with these cognitive difficulties in long COVID. Therefore, there is no data to suggest which strategy would help these patients.

## Trial status

The study is currently enrolling patients. The first randomisation was carried out on 07 April 2022 and the last randomization should take place in August 2023. Protocol version: 1.4 (reference of the ethics committee: 2021/432—27/02/2023).

## Data Availability

The dataset generated during the trial will be available upon reasonable request from the CI. The data will be available following publication of the trial results.

## References

[1] Jiang L, Tang K, Levin M, Irfan O, Morris SK, Wilson K (2020). COVID-19 and multisystem inflammatory syndrome in children and adolescents. Lancet Infect Dis.

[2] Harapan BN, Yoo HJ (2021). Neurological symptoms, manifestations, and complications associated with severe acute respiratory syndrome coronavirus 2 (SARS-CoV-2) and coronavirus disease 19 (COVID-19). J Neurol..

[3] Miskowiak KW, Johnsen S, Sattler SM, Nielsen S, Kunalan K, Rungby J (2021). Cognitive impairments four months after COVID-19 hospital discharge: Pattern, severity and association with illness variables. Eur Neuropsychopharmacol.

[4] Zhou H, Lu S, Chen J, Wei N, Wang D, Lyu H (2020). The landscape of cognitive function in recovered COVID-19 patients. J Psychiatr Res.

[5] Méndez R, Balanzá-Martínez V, Luperdi SC, Estrada I, Latorre A, González-Jiménez P (2022). Long-term neuropsychiatric outcomes in COVID-19 survivors: A 1-year longitudinal study. J Intern Med.

[6] Taquet M, Geddes JR, Husain M, Luciano S, Harrison PJ (2021). 6-month neurological and psychiatric outcomes in 236 379 survivors of COVID-19: a retrospective cohort study using electronic health records. Lancet Psychiatry.

[7] Ferrucci R, Dini M, Groppo E, Rosci C, Reitano MR, Bai F (2021). Long-lasting cognitive abnormalities after COVID-19. Brain Sci.

[8] Poletti S, Palladini M, Mazza MG, De Lorenzo R, Furlan R, Ciceri F, et al. Long-term consequences of COVID-19 on cognitive functioning up to 6 months after discharge: role of depression and impact on quality of life The COVID-19 BioB Outpatient Clinic Study group. Eur Arch Psychiatry Clin Neurosci. 2022;272:773–82. 10.1007/s00406-021-01346-9 PMC8546751 34698871

[9] Nalbandian A, Sehgal K, Gupta A, Madhavan MV, McGroder C, Stevens JS (2021). Post-acute COVID-19 syndrome. Nat Med.

[10] Boldrini M, Canoll PD, Klein RS (2021). How COVID-19 Affects the Brain. JAMA Psychiat.

[11] Peiris S, Mesa H, Aysola A, Manivel J, Toledo J, Borges-Sa M (2021). Pathological findings in organs and tissues of patients with COVID-19: A systematic review. PLoS ONE.

[12] Hosp JA, Dressing A, Blazhenets G, Bormann T, Rau A, Schwabenland M (2021). Cognitive impairment and altered cerebral glucose metabolism in the subacute stage of COVID-19. Brain.

[13] Guedj E, Lazarini F, Morbelli S, Ceccaldi M, Hautefort C, Kas A, Radulesco T, Salmon-Ceron D, Eldin C (2021). Long COVID and the brain network of Proust's madeleine: targeting the olfactory pathway. Clin Microbiol Infect.

[14] Parsons N, Outsikas A, Parish A, Clohesy R, D’Aprano F, Toomey F (2021). Modelling the Anatomic Distribution of Neurologic Events in Patients with COVID-19: A Systematic Review of MRI Findings. Am J Neuroradiol.

[15] Toniolo S, Di Lorenzo F, Scarioni M, Frederiksen KS, Nobili F (2021). Is the Frontal Lobe the Primary Target of SARS-CoV-2?. J Alzheimers Dis.

[16] Yesilkaya UH, Balcioglu YH (2020). Neuroimmune correlates of the nervous system involvement of COVID-19: A commentary. J Clin Neurosci.

[17] Ceban F, Ling S, Lui LMW, Lee Y, Gill H, Teopiz KM (2022). Fatigue and cognitive impairment in Post-COVID-19 Syndrome: A systematic review and meta-analysis. Brain Behav Immun.

[18] Diana L, Regazzoni R, Sozzi M, Piconi S, Borghesi L, Lazzaroni E (2023). Monitoring cognitive and psychological alterations in COVID-19 patients: A longitudinal neuropsychological study. J Neurol Sci.

[19] Rohling ML, Faust ME, Beverly B, Demakis G (2009). Effectiveness of Cognitive Rehabilitation Following Acquired Brain Injury: A Meta-Analytic Re-Examination of Cicerone et al.'s (2000, 2005) Systematic Reviews. Neuropsychology..

[20] Ponsford J, Velikonja D, Janzen S, Harnett A, McIntyre A, Wiseman-Hakes C (2023). INCOG 2.0 Guidelines for Cognitive Rehabilitation Following Traumatic Brain Injury, Part II: Attention and Information Processing Speed. J Head Trauma Rehabil..

[21] Velikonja D, Ponsford J, Janzen S, Harnett A, Patsakos E, Kennedy M (2023). INCOG 2.0 Guidelines for Cognitive Rehabilitation Following Traumatic Brain Injury, Part V: Memory. J Head Trauma Rehabil.

[22] Jeffay E, Ponsford J, Harnett A, Janzen S, Patsakos E, Douglas J (2023). INCOG 2.0 Guidelines for Cognitive Rehabilitation Following Traumatic Brain Injury, Part III: Executive Functions. J Head Trauma Rehabil..

[23] Palladini M, Bravi B, Colombo F, Caselani E, Di Pasquasio C, D’Orsi G, et al. Cognitive remediation therapy for post-acute persistent cognitive deficits in COVID-19 survivors: A proof-of-concept study. Neuropsychol Rehabil. 2022;33(7):1–18. 10.1080/09602011.2022.2075016 35583357

[24] Gómez-De-Regil L, Estrella-Castillo DF, Vega-Cauich J (2019). Psychological Intervention in Traumatic Brain Injury Patients. Behav Neurol.

[25] Mittenberg W, Tremont G, Zielinski RE, Fichera S, Rayls KR (1996). Cognitive-behavioral prevention of postconcussion syndrome. Arch Clin Neuropsychol.

[26] Silverberg ND, Hallam BJ, Rose A, Underwood H, Whitfield K, Thornton AE, Whittal ML (2013). Cognitive-behavioral prevention of postconcussion syndrome in at-risk patients: a pilot randomized controlled trial. J Head Trauma Rehabil.

[27] Huang L, Yao Q, Gu X, Wang Q, Ren L, Wang Y, Hu P, Guo L, Liu M, Xu J, Zhang X (2021). 1-year outcomes in hospital survivors with COVID-19: a longitudinal cohort study. The Lancet.

[28] Working Group to Develop a Clinician’s Guide to Cognitive Rehabilitation in mTBI: Application for Military Service Members and Veterans. Clinician’s guide to cognitive rehabilitation in mild traumatic brain injury: Application for military service members and veterans. Rockville, MD: American Speech-Language-Hearing Association. 2016. Available from http://www.asha.org/uploadedFiles/ASHA/Practice_Portal/Clinical_Topics/Traumatic_Brain_Injury_in_Adults/Clinicians-Guide-to-Cognitive-Rehabilitation-in-Mild-Traumatic-Brain-Injury.pdf .

[29] Winkens I, Van Heugten CM, Wade DT, Habets EJ, Fasotti L (2009). Efficacy of Time Pressure Management in Stroke Patients With Slowed Information Processing: A Randomized Controlled Trial. Arch Phys Med Rehabil.

[30] Wade SL, Walz NC, Carey J, McMullen KM, Cass J, Mark E, Yeates KO (2012). A randomized trial of teen online problem solving: efficacy in improving caregiver outcomes after brain injury. Health Psychol.

[31] Helmick K (2010). Cognitive rehabilitation for military personnel with mild traumatic brain injury and chronic post-concussional disorder: Results of April 2009 consensus conference. NeuroRehabilitation.

[32] Levine B, Schweizer TA, O'Connor C, Turner G, Gillingham S, Stuss DT, Manly T, Robertson IH (2011). Rehabilitation of executive functioning in patients with frontal lobe brain damage with goal management training. Frontiers Human Neuroscience.

[33] Sohlberg MM, Kennedy M, Avery J, Coelho C, Turkstra L, Ylvisaker M, et al. Evidence-based practice for the use of external aids as a memory compensation technique. J Med Speech Lang Pathol. 2007;15(1):xv–li.

[34] Wilson B, Emslie H, Quirk K, Evans J, Injury PWB, 2005 undefined (2005). A randomized control trial to evaluate a paging system for people with traumatic brain injury. Taylor & Francis..

[35] Seron X, Van der Linden, M. Traité de neuropsychologie clinique de l’adulte: Tome 2-Rééducation. Paris: Deboeck supérieur; 2016. p. 512.

[36] Kaplan SJ. The private practice of behavior therapy: A guide for behavioral practitioners. Berlin: Springer; 2013. p. 288.

[37] Fanget F, Rouchouse B. Affirmation de soi (L’): Une méthode de thérapie. Paris: Odile Jacob; 2007. p. 368.

[38] Langlois F, Gosselin P, Brunelle C, Drouin MC, Ladouceur R (2007). Les variables cognitives impliquées dans l'inquiétude face à la maladie. Can J Behav Sci.

[39] Waid-Ebbs JK, Wen PS, Heaton SC, Donovan NJ, Velozo C (2012). The item level psychometrics of the behaviour rating inventory of executive function-adult (BRIEF-A) in a TBI sample. Brain Inj.

[40] Troyer AK, Rich JB (2002). Psychometric Properties of a New Metamemory Questionnaire for Older Adults. J Gerontol B Psychol Sci Soc Sci.

[41] Duquette RL, Dupuis G, Perrault J (1994). A new approach for quality of life assessment in cardiac patients: rationale and validation of the Quality of Life Systemic Inventory. Can J Cardiol.

[42] Brooks R, Group E (1996). EuroQol: the current state of play. Health Policy.

[43] Zimmermann P, Fimm B (2002). A test battery for attentional performance. Applied neuropsychology of attention. Theory, diagnosis and rehabilitation..

[44] Brickenkamp R, Schmidt-Atzert L, Liepmann D. D2-R: test d’attention concentrée-révisé: manuel. Paris: Hogrefe; 2015. p. 108.

[45] Azouvi P, Vallat-Azouvi C, Joseph PA, Meulemans T, Bertola C, Le Gall D, Bellmann A, Roussel M, Coyette F, Krier M, Franconie C (2016). Executive functions deficits after severe traumatic brain injury: The GREFEX study. J head trauma rehabil.

[46] Geurten M, Vincent E, Van Der Linden M, Meulemans T (2016). Working memory assessment: Construct validity of the brown-peterson test. Can J Behav Sci.

[47] Randolph C, Tierney MC, Mohr E, Chase TN (1998). The Repeatable Battery for the Assessment of Neuropsychological Status (RBANS): preliminary clinical validity. J Clin Exp Neuropsychol.

[48] Benedict RHB, Groninger L, Schretlen D, Dobraski M, Shpritz B (1996). Revision of the brief visuospatial memory test: Studies of normal performance, reliability, and validity. Psychol Assess.

[49] Nasreddine ZS, Phillips NA, Bédirian V, Charbonneau S, Whitehead V, Collin I (2005). The Montreal Cognitive Assessment, MoCA: A brief screening tool for mild cognitive impairment. J Am Geriatr Soc.

[50] Buysse DJ, Reynolds CF, Monk TH, Hoch CC, Yeager AL, Kupfer DJ (1991). Quantification of Subjective Sleep Quality in Healthy Elderly Men and Women Using the Pittsburgh Sleep Quality Index (PSQI). Sleep.

[51] Fisk JD, Pontefract A, Ritvo PG, Archibald CJ, Murray TJ (1994). The impact of fatigue on patients with multiple sclerosis. Cambridge org..

[52] Simon W, Lambert MJ, Harris MW, Busath G, Simon W, Lambert MJ, et al. Providing patient progress information and clinical support tools to therapists : Effects on patients at risk of treatment failure. 2012;3307:638–47. 10.1080/10503307.2012.698918 22755547

[53] Reilly MC, Zbrozek AS, Dukes EM (1993). The Validity and Reproducibility of a Work Productivity and Activity Impairment Instrument. Pharmacoeconomics.

[54] Kang H. Sample size determination and power analysis using the G*Power software. J Educ Eval Health Prof. 2021;18:17. 10.3352/jeehp.2021.18.17 PMC8441096 34325496

[55] Audrit H, Beauchamp MH, Tinawi S. Multidimensional Psychoeducative and Counseling Intervention (SAAM) for Symptomatic Patients With Mild Traumatic Brain Injury : A Pilot Randomized Controlled Trial. 2021;36(4):249–61. 10.1097/HTR.0000000000000653 33656475

[56] Scheenen ME, Visser-Keizer AC, de Koning ME, van der Horn HJ, van de Sande P, van Kessel M, van der Naalt J, Spikman JM (2017). Cognitive behavioral intervention compared to telephone counseling early after mild traumatic brain injury: a randomized trial. J Neurotrauma.

[57] Prince C, Bruhns ME (2017). Evaluation and treatment of mild traumatic brain injury: the role of neuropsychology. Brain Sci.

[58] Gouraud C, Bottemanne H, Lahlou-Laforêt K, Blanchard A, Günther S, Batti S El, et al. Association Between Psychological Distress, Cognitive Complaints, and Neuropsychological Status After a Severe COVID-19 Episode: A Cross-Sectional Study. Front Psychiatry. 2021;12:725861. 10.3389/fpsyt.2021.725861 PMC8446522 34539470

[59] Potter SD, Brown RG, Fleminger S (2016). Randomised, waiting list controlled trial of cognitive–behavioural therapy for persistent postconcussional symptoms after predominantly mild–moderate traumatic brain injury. J Neurol Neurosurg Psychiatry.

