# Correction: COVCOG: Immediate and long-term cognitive improvement after cognitive versus emotion management psychoeducation programs—a randomized trial in covid patients with neuropsychological difficulties

**DOI:** 10.1186/s12883-023-03372-7

**Published:** 2023-09-11

**Authors:** Sylvie Willems, Vincent Didone, Carmen Cabello Fernandez, Gael Delrue, Hichem Slama, Patrick Fery, Julien Goin, Clara Della Libera, COVCOG Group, Fabienne Collette

**Affiliations:** 1 Psychology and Neuroscience of Cognition Unit, Université de Liège, Place Des Orateurs, 1, B33 4000 Liège, Belgium; 2 University Psychology and Speech Therapy Clinic, CPLU, Université de Liège, Liège, Belgium; 3 Clinical Neuropsychological Unit, Liège University Hospital, CHU de Liège, Liège, Belgium; 4 Clinical Neuropsychological Unit, Brussel University Hospital, Erasme, Brussels, Belgium; 5 GIGA-CRC, Université de Liège and Belgian National Fund for Scientific Research, In Vivo Imaging, Liège, Belgium

**Correction: BMC Neurol 23, 307 (2023)**

**https://doi.org/10.1186/s12883-023-03346-9**

Following publication of the original article [1], the authors identified an error in the author name of Fabienne Collette. The given name and family name were erroneously transposed.

The incorrect author name is: Collette Fabienne

The correct author name is: Fabienne Collette

The author group has been updated above and the original article [1] has been corrected.
